# FancJ regulates interstrand crosslinker induced centrosome amplification through the activation of polo-like kinase 1

**DOI:** 10.1242/bio.20135801

**Published:** 2013-08-06

**Authors:** Jianqiu Zou, Fen Tian, Ji Li, Wyatt Pickner, Molly Long, Khosrow Rezvani, Hongmin Wang, Dong Zhang

**Affiliations:** Basic Biomedical Science Division, Sanford School of Medicine, University of South Dakota, Vermillion, South Dakota, 57069, USA

**Keywords:** FancJ, Centrosome, PLK1

## Abstract

DNA damage response (DDR) and the centrosome cycle are two of the most critical processes for maintaining a stable genome in animals. Sporadic evidence suggests a connection between these two processes. Here, we report our findings that six Fanconi Anemia (FA) proteins, including FancI and FancJ, localize to the centrosome. Intriguingly, we found that the localization of FancJ to the mother centrosome is stimulated by a DNA interstrand crosslinker, Mitomycin C (MMC). We further show that, in addition to its role in interstrand crosslinking (ICL) repair, FancJ also regulates the normal centrosome cycle as well as ICL induced centrosome amplification by activating the polo-like kinase 1 (PLK1). We have uncovered a novel function of FancJ in centrosome biogenesis and established centrosome amplification as an integral part of the ICL response.

## Introduction

The centrosome is an important organelle and functions primarily to organize microtubules (MT) in animal cells and serve as the platform for the regulation of a variety of signaling pathways (Nigg and Raff, 2009; Nigg and Stearns, 2011). Most animal cells have either one or two centrosomes depending on the cell cycle stage. In a proliferating animal cell, the number and structure of centrosomes are highly regulated during each cell cycle. Similar to the replication of DNA, the centrosome duplicates once, and only once, per cell cycle. The number and integrity of the centrosomes can greatly impact the accuracy of chromosome segregation. For example, Pellman and colleagues recently provided strong evidence linking extra centrosomes and chromosomal instability (CIN) (Ganem et al., 2009). They showed that, even though the majority of cells with extra centrosomes still undergo bipolar cell division, likely through centrosome clustering (Quintyne et al., 2005), the extra centrosomes alone are sufficient to promote chromosome missegregation due to increased frequency of merotelic kinetochore attachments in which a single kinetochore might capture microtubules from multiple centrosomes. Therefore, the centrosome cycle is critical for genome stability.

Another important cellular process affecting genome stability is DNA damage response (DDR) (Ciccia and Elledge, 2010). In response to genotoxic stress, cells launch a series of signaling and repair responses that are collectively called DDR. Two groups of kinases play critical roles during the DDR: (1) PI-3 kinase-like kinases (PIKKs), including ATM, ATR, and DNA-PK; and (2) checkpoint kinases, including Chk1, Chk2, and MAPKAP kinase-2, all of which are serine/threonine kinases. Checkpoint kinases are activated by the PIKKs that are facilitated by a group of checkpoint mediator proteins, including BRCA1, MDC1, Claspin, and TopBP1. Downstream of these early DDR factors are a variety of DNA repair pathways for repairing different forms of genotoxic lesions (Friedberg, 2003).

Intriguingly, it has been observed that a few DDR proteins localize to the centrosome (supplementary material Table S1) and a variety of genotoxic stress can induce dramatic centrosome amplification (DNA damage-induced centrosome amplification, DDICA) (Balczon et al., 1995; Sibon et al., 2000). Compared to the regulation of a normal centrosome cycle, DDICA is much less understood. Nonetheless, DDICA is thought to be a fail-safe mechanism for eliminating cells with extensive DNA damage (Nigg and Stearns, 2011). For example, when syncytial Drosophila embryos are treated with a variety of DNA damaging agents, including ionizing radiation (IR), which induces double-stranded breaks (DSB), and Aphidicolin and hydroxyurea (HU), both of which block DNA replication, centrosome amplification occurs in cells experiencing severe DNA damage. These cells are subsequently eliminated from the embryo (Sibon et al., 2000). In Drosophila, DDICA is dependent on Chk2 (Takada et al., 2003). DDICA is also observed in certain cultured mammalian cells, including U2-OS and CHO cells (Balczon et al., 1995; Liu and Erikson, 2002). Proteins involved in regulating DDICA include PLK1, Chk1, MDC1, and BRIT1/MCPH1 (Bourke et al., 2007; Inanç et al., 2010; Liu and Erikson, 2002; Löffler et al., 2006; Lončarek et al., 2010; Rai et al., 2008). However, the detailed molecular mechanism behind DDICA is still a mystery.

PLK1 is a canonical serine/threonine kinase and belongs to the family of polo-like kinases (Archambault and Glover, 2009; de Cárcer et al., 2011; Lens et al., 2010; Strebhardt, 2010). PLK1 is highly conserved, even among divergent species such as yeast, Drosophila, Xenopus and mammals. PLK1 contains two conserved domains: an N-terminal kinase catalytic domain and a C-terminal polo box domain (PBD). The PBD domain is implicated in associating with the substrates of PLK1, and often requires priming phosphorylation by cyclin-dependent kinases (CDK). PLK1 regulates a variety of biological processes during G2, G2 to M transition, and M phase. As related to centrosome biogenesis, PLK1 is required for centriole disengagement in late mitosis, which is an essential licensing step for the next centrosome cycle (Tsou et al., 2009). Likely because of this function, PLK1 is also critical for DDICA (Inanç et al., 2010; Liu and Erikson, 2002; Lončarek et al., 2010). Recently, Aurora A was identified as the upstream activating kinase of PLK1. The kinase activity of Aurora A is stimulated by a cofactor, hBora (Macůrek et al., 2008; Seki et al., 2008). Although not absolutely required for the normal G2 to M transition, PLK1 becomes essential for the G2 to M transition during the DNA damage recovery process (van Vugt et al., 2004). The activation of PLK1 by Aurora A and hBora is required for its function during DNA damage recovery (Macůrek et al., 2008). Whether Aurora A and hBora are also involved in the centrosome cycle function of PLK1 is currently unknown.

To further define the interconnection between DDR and the centrosome, we performed a focused immunofluorescence screening using over 100 antibodies against 44 DDR proteins in order to identify which one(s) localizes to the centrosome. Among the many interesting findings, we discovered that the products of six Fanconi Anemia (FA) related genes (out of 10 tested), including FancJ, localize at the centrosome. FA proteins have well established functions in repairing interstrand crosslinking (ICL) lesions (Kee and D'Andrea, 2010). A few studies showed that deficiency of some of the FA genes also induce centrosome amplification (Collis et al., 2008; Katsura et al., 2009; Tutt et al., 1999). However, their functions in the centrosome cycle are unknown. We show here, using immunofluorescence analysis, that FancJ localizes to the centrosome and has unique centrosome staining patterns. In addition, the centrosome localization of FancJ is regulated by an ICL inducer, Mitomycin C (MMC). Similar to HU and IR, we found that ICL inducers, including MMC and cis-platin, also induce pronounced centrosome amplification. Most importantly, we show that FancJ promotes the MMC-induced centrosome amplification by activating PLK1. These data strongly implicate FancJ in the regulation of normal centrosome cycle as well as DDICA.

## Results

### Immunofluorescence screening shows that six Fanconi Anemia proteins localize to the centrosome

**Fig. 1.**
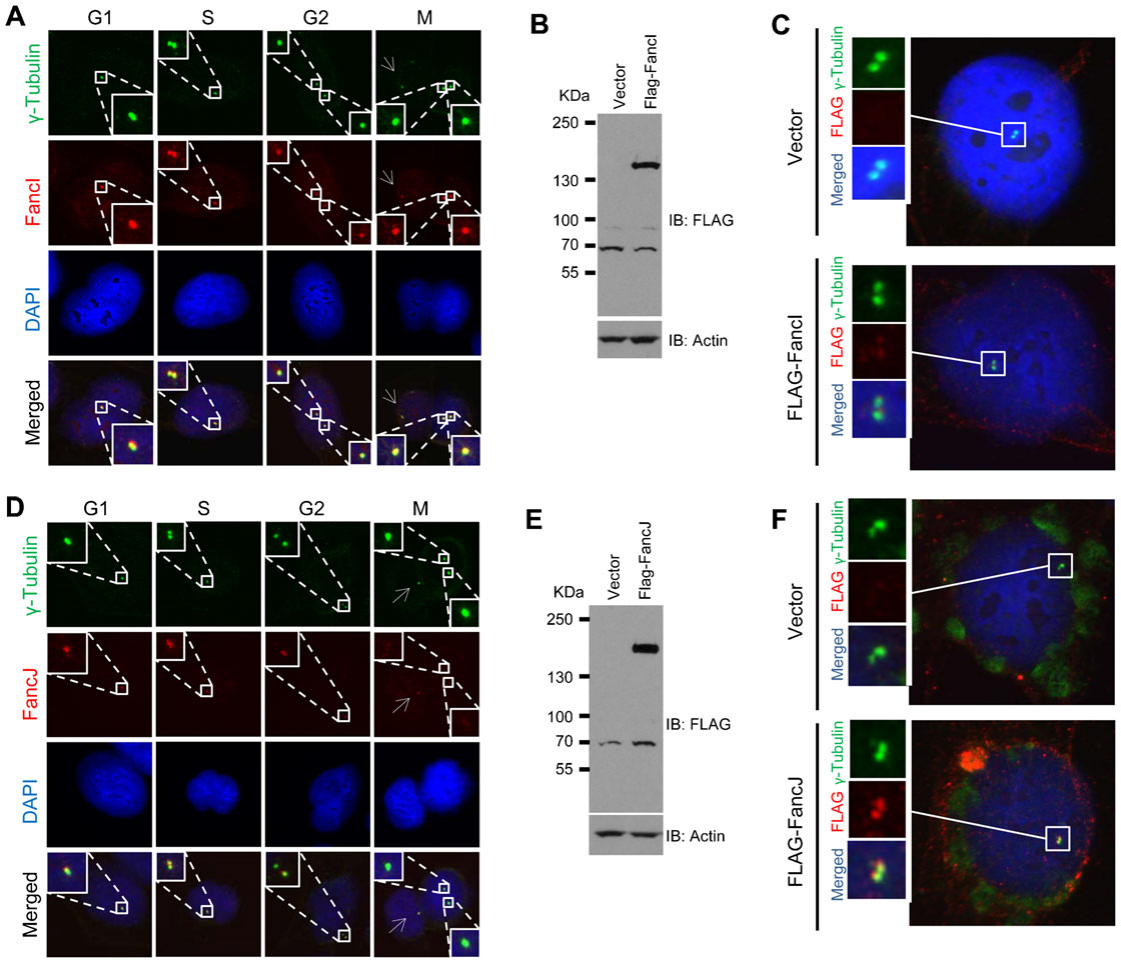
Two Fanconi Anemia (FA) related proteins, FancI and FancJ, localize to the centrosome. U2-OS cells were fixed in methanol and co-stained with antibodies against γ-Tubulin (green) and FancI (**A**) (red) or FancJ (**D**) (red) as indicated. Nuclei were stained with DAPI (blue). In the column of the mitotic cell, the arrow indicates an interphase centrosome. (**B**,**C**) U2-OS cells were transiently transfected with either vector or FLAG-tagged FancI and split into two sets. One set of cells was used for Western Blot analysis to monitor protein expression (B). Antibodies used for immunoblotting are indicated on the right. The second set of cells was fixed in methanol and stained with antibody against γ-Tubulin (green) and FLAG (red). Nuclei were stained with DAPI (blue) (C). (**E**,**F**) U2-OS cells were transiently transfected with either vector or FLAG-tagged FancJ and split into two sets. One set of cells was used for Western Blot analysis to monitor protein expression (E). Antibodies used for immunoblotting are indicated on the right. The second set of cells was fixed in methanol and stained with antibody against γ-Tubulin (green) and FLAG (red) (F). Nuclei were stained with DAPI (blue).

To further define the interconnection between DDR and the centrosome, we performed a focused immunofluorescence screening in U2-OS cells using over 100 antibodies against 44 DDR proteins in order to identify which one(s) localizes to the centrosome. γ-Tubulin was used as the marker for the centrosome. From this screening, we detected the products of six Fanconi Anemia (FA) related genes (out of ten tested) at the centrosome (Fig. 1; supplementary material Figs S1, S2). We detected two of the FA factors, FancI and FancM, at the centrosome with multiple antibodies (Fig. 1; supplementary material Fig. S2). We further validated the centrosome staining of FancI and FancJ by expressing FLAG-tagged FancI or FancJ and staining cells with anti-FLAG antibody (Fig. 1B,C,E,F). Additionally, we demonstrated that small inference RNA (siRNA) depletion of FancI or FancJ dramatically reduced their centrosome staining (supplementary material Fig. S3). More interestingly, some of the FA factors have very unique centrosome staining patterns (Fig. 1; supplementary material Fig. S1). Based on their staining patterns, we classified the six FA genes into three different groups. Group I: Stain the centrosome throughout the cell cycle. Cells in G1 and early S phase have one centrosome, as indicated by one γ-Tubulin staining dot. Late S and early G2 phase cells have two closely positioned γ-Tubulin dots. Cells in late G2 and throughout mitosis have two further separated γ-Tubulin dots. FancG is an example of this group, which also includes Fanc-B, -I, and -M. Group II: FancA shows positive centrosome staining in G1, S, and early G2 phase. However, during late G2 and mitosis, FancA mainly localizes to one of the two separated centrosomes in 80% of the cells (supplementary material Fig. S1D). We therefore call this staining pattern “asymmetrical centrosome staining”. In order to distinguish the mother centriole from the daughter centriole, we co-stained cells with a newly identified mother centriole marker, Chibby (Steere et al., 2012). As seen in supplementary material Fig. S1E, in the large majority of cells, FancA colocalizes with Chibby suggesting that FancA primarily localizes to the mother centriole. Group III: FancJ shows positive centrosome staining in G1, S, and early G2 phase. However, its centrosomal staining is dramatically reduced during late G2 or mitosis (note the dramatically reduced FancJ staining in the two separated centrosomes in Fig. 1D). In a small percentage of cells, FancJ weakly stains one of the two separated centrosome (Fig. 1D, Fig. 4B). Recently, Boulton and colleagues showed that siRNA depletion of FancM induces centrosome amplification, suggesting that FancM may play a role in regulating the centrosome cycle (Collis et al., 2008). Consistent with their findings, we found that three different FancM antibodies positively stain the centrosome (supplementary material Fig. S2).

### FancJ, but not FancI, regulates normal centrosome cycle and HU-induced centrosome amplification

**Fig. 2.**
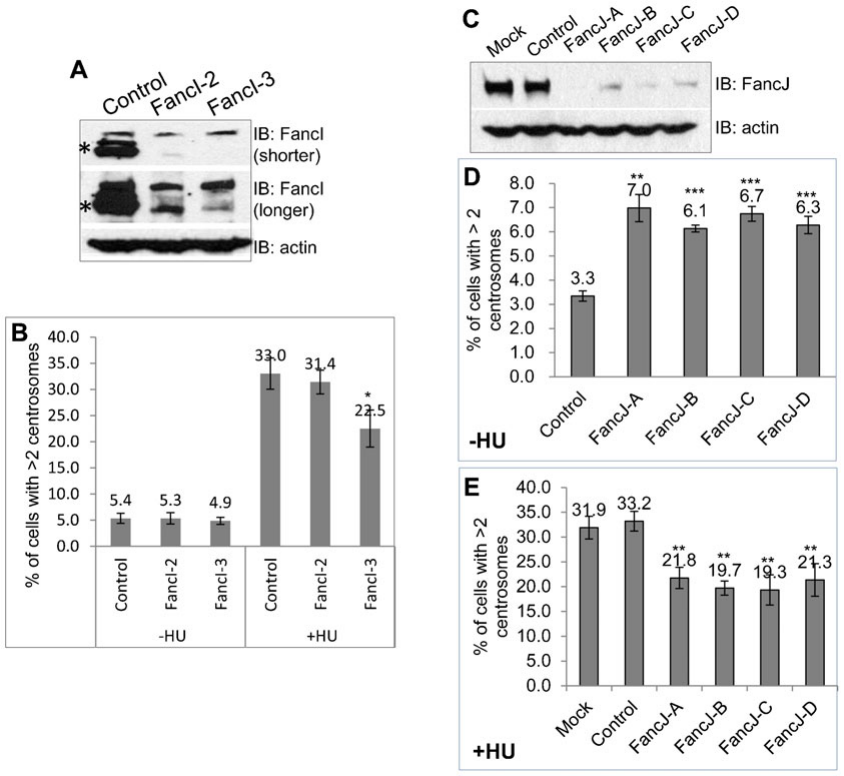
Deficiency of FancJ, but not FancI, affects centrosome biogenesis. U2-OS cells were transfected with either Control siRNA, or siRNA against FancI (**A**,**B**), or FancJ (**C–E**) and then split into three sets. One set of cells was used for Western Blot analysis to monitor the siRNA knockdown efficiency. Antibodies used for immunoblotting are indicated on the right (A,C). The second set of cells was fixed in methanol and stained with antibody against γ-Tubulin (B,D). The third set of cells was first treated with 16 mM HU for 68 hours and then fixed in methanol and stained with antibody against γ-Tubulin (B,E). More than 300 cells were counted and the percentage of cells with more than two centrosomes was quantitated (B,D,E). * indicates the band corresponding to FancI. Mock refers to cells without siRNA transfection. All error bars are standard deviation obtained from three different experiments. Standard two-sided t test, *P<0.05, **P<0.01, ***P<0.001.

Since the FA pathway was well represented at the centrosome, we focused on the proteins in this pathway. First, we looked to see if any of the FA proteins regulates the centrosome number. We used siRNA to deplete two of the FA proteins that stained positive at the centrosome, FancI, and FancJ. The siRNA transfected cells were then stained with antibody against γ-Tubulin to mark the centrosome and the percentage of cells with more than two centrosomes was then quantitated. As shown in Fig. 2A,B, depletion of FancI does not affect the centrosome number in U2-OS cells. Interestingly, depletion of FancJ with four different siRNA doubled the number of cells with more than two centrosomes in both U2-OS and Hs587T cells (Fig. 2C–E; supplementary material Fig. S4A,B), suggesting that FancJ may play a role in the regulation of normal centrosome cycle. The centrosome amplification in FancJ depleted cells is less likely to be caused by DNA damage because there is no detectable γ-H2AX focus in FancJ siRNA transfected cells (supplementary material Fig. S5). Another long-standing observation related to centrosome biogenesis is that prolonged S phase arrest in certain mammalian cells with DNA replication blockers, including Aphidicolin and HU, induces pronounced centrosome amplification (Balczon et al., 1995). Although the exact biological implication of this phenomenon is still unknown, it is proposed to be a fail-safe mechanism to eliminate cells with extensive DNA damage (Nigg and Stearns, 2011). We found that only one of the siRNAs targeting FancI (siRNA-FancI-3) mildly reduced the HU-induced centrosome amplification (Fig. 2B). We noted that siRNA-FancI-3 knocked down FancI more efficiently than siRNA-FancI-2 (Fig. 2A). Intriguingly, depletion of FancJ with four different siRNA reduced 40% of HU induced centrosome amplification (Fig. 2E; supplementary material Fig. S4C), suggesting that FancJ is also involved in regulating the DDICA. This decrease is not due to any cell cycle changes in FancJ siRNA depleted cells (supplementary material Fig. S6). Taken together, these data suggest that in addition to its well established checkpoint and DNA repair functions (Cantor et al., 2001; Hiom, 2010), FancJ also plays an important role in centrosome biogenesis.

### Interstrand crosslinking agents induce PLK1-dependent centrosome amplification

**Fig. 3.**
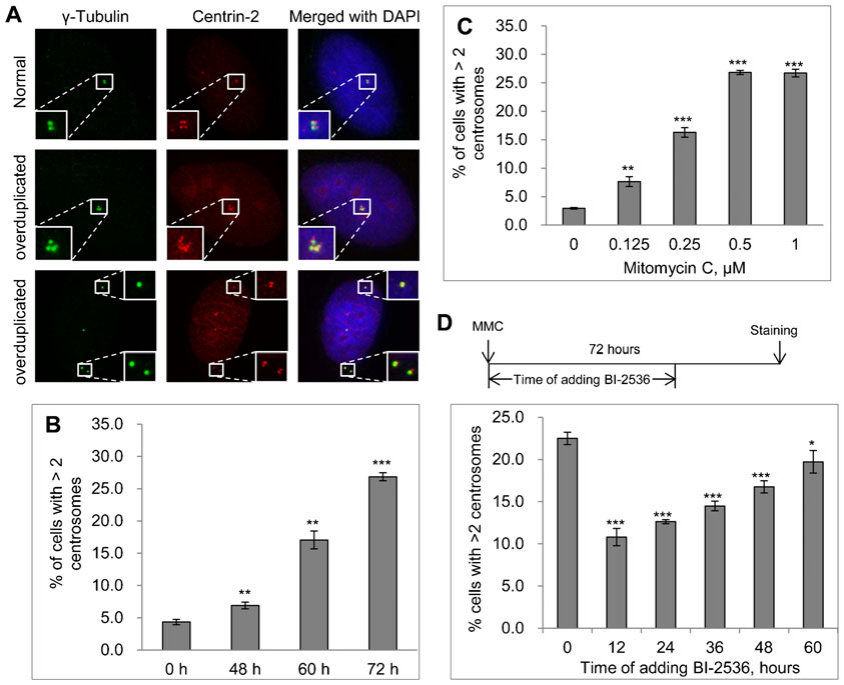
ICL agent, MMC induces pronounced centrosome amplification. (**A**) U2-OS cells were treated with 0.5 µM of MMC, fixed in methanol and then stained with antibodies against γ-Tubulin (green) and Centrin-2 (red). Nuclei were stained with DAPI (blue). (**B**) Time-course experiment. U2-OS cells were treated with 0.5 µM of MMC. At the indicated time, cells were fixed in methanol and stained with antibody against γ-Tubulin. (**C**) Dose response experiment. 72 hours after treatment with the indicated concentration of MMC, U2-OS cells were fixed in methanol and stained with antibody against γ-Tubulin. (**D**) Inhibition of MMC induced centrosome amplification by PLK1 kinase inhibitor, BI-2536. U2-OS cells were treated with 0.5 µM of MMC for 72 hours. At the indicated time, either no BI-2536 was added (0), or 100 nM BI-2536 was added. Cells were fixed in methanol and stained with antibody against γ-Tubulin. In all the experiments, more than 300 cells were counted and the percentage of cells with more than two centrosomes was quantitated. All error bars are standard deviation obtained from three different experiments. Standard two-sided t test, *P<0.05, **P<0.01, ***P<0.001.

Because the major function of FA proteins is to repair ICL lesions, we next tested whether ICL agents can induce centrosome amplification. We first performed a time-course experiment. U2-OS cells were treated with 0.5 µM MMC for 48, 60, or 72 hours and then stained with anti-γ-Tubulin antibody to mark the centrosome and anti-Centrin-2 antibody to mark the centriole. Interestingly, similar to HU and IR, MMC also induced pronounced centrosome amplification. Fig. 3A shows representative images of MMC-treated cells with a normal centrosome (top panel) and with amplified centrosomes (middle and bottom panels). Forty-eight hour treatment with MMC mildly induced centrosome amplification. Interestingly, treatment of U2-OS cells with MMC for 60 or 72-hr induced 4-fold and 7-fold centrosome amplification, respectively (Fig. 3B), which is comparable to the amount of centrosome amplification induced by HU (Fig. 2B,E). Next, we performed an MMC dose response experiment. We treated U2-OS cells with different concentrations of MMC for 72 hours before the cells were stained with anti-γ-Tubulin antibody. As shown in Fig. 3C, as low as 0.125 µM MMC induced significant centrosome amplification. The concentration of MMC that induced the maximum centrosome amplification was 0.5 µM. In addition, similar to MMC, another ICL inducer, cis-platin, also induced robust centrosome amplification (supplementary material Fig. S7), suggesting that centrosome amplification is likely an integral part of the ICL induced response.

One of the known factors important for DDICA is PLK1 (Inanç et al., 2010; Liu and Erikson, 2002; Lončarek et al., 2010). Next, we tested whether MMC-induced centrosome amplification is also dependent on PLK1 using a potent PLK1 inhibitor BI-2536 (Steegmaier et al., 2007). BI-2536 was added at different times after the addition of MMC (Fig. 3D). BI-2536 reduced MMC-centrosome amplification by about 50% following 12 or 24 hour treatment with MMC. Interestingly, if BI-2536 was added at 48 or 60 hours to MMC treated cells, it had very little effect on the MMC-induced centrosome amplification. Two interesting conclusions can be drawn from this experiment. First, similar to its role in HU- and IR-induced centrosome amplification, the activity of PLK1 is also critical for MMC-induced centrosome amplification. Second, PLK1 is probably activated around 24 to 48 hours after MMC addition and functions during that period of time. After that, the activity of PLK1 is no longer needed. It was previously shown that DNA damage inhibits PLK1 activity (Smits et al., 2000). Other evidence, plus the data presented here, indicate that certain PLK1 activity is needed for DNA damage induced centrosome amplification (Inanç et al., 2010; Liu and Erikson, 2002; Lončarek et al., 2010). Therefore, it appears that not all PLK1 is inhibited by DNA damage. Alternatively, a fraction of PLK1 may be reactivated at a later time after DNA damage. Currently, we do not know which of these scenarios is true.

To distinguish mother centriole from daughter centriole in MMC treated cells, we also stained them with the mother centriole maker, Chibby (Steere et al., 2012). For a normal centrosome, one γ-Tubulin dot should match two Centrin-2 dots (Fig. 3A; supplementary material Fig. S8A) and one Chibby dot should match four Centrin-2 dots (supplementary material Fig. S1E, Fig. S8C). Using these rules, we tentatively classified cells with amplified centrosomes into three groups (supplementary material Fig. S8): Type 1 had more γ-Tubulin or Chibby dots than Centrin-2 dots; Type 2 had an equal number of γ-Tubulin or Chibby dots and Centrin-2 dots; Type 3 had less γ-Tubulin or Chibby dots than Centrin-2 dots. Type 1 is potentially caused by premature assembly of PCM or dysregulated maturation of the centriole. Type 2 represents a bona fide amplified centrosome. Type 3 could be caused by hyper-amplification of centriole or centriole fragmentation. Most of MMC induced centrosome amplification seems to fall into Type 1 and Type 3.

Together with previous findings, our data indicate that the DNA damage-induced centrosome amplification is likely to be an integral part of the broader DNA damage response.

### MMC regulates the centrosome localization of FancJ while FancJ stimulates MMC induced centrosome amplification

**Fig. 4.**
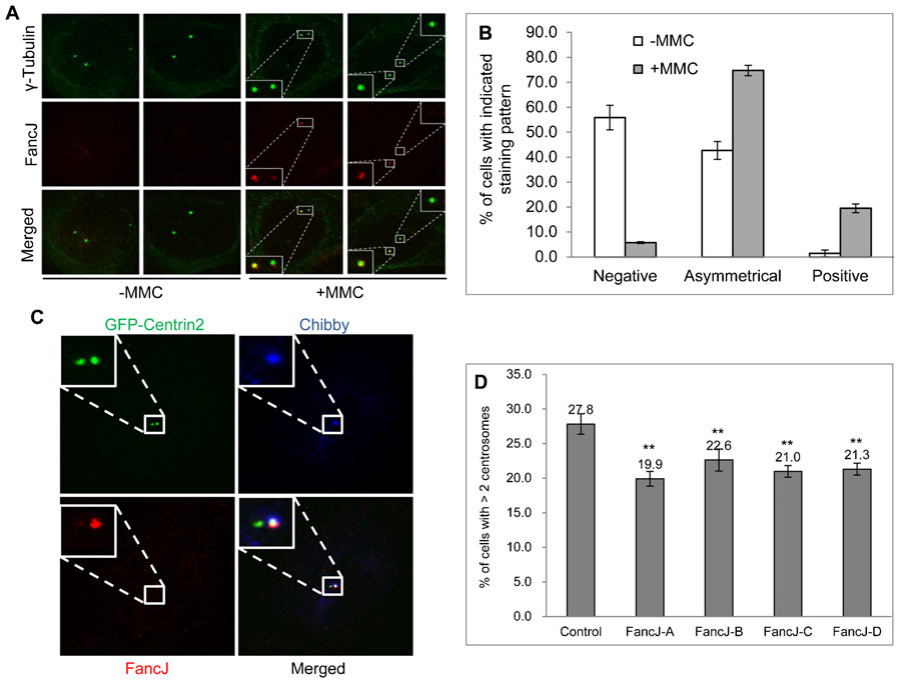
FancJ promotes MMC induced centrosome amplification. MMC enhances FancJ centrosome localization in G2 or M cells (**A–C**). U2-OS cells were either left untreated (−MMC) or treated with 0.5 µM of MMC overnight. Cells were then fixed in methanol and stained with antibodies against γ-Tubulin (green) and FancJ (red). Representative images are shown (A). More than 100 cells with G2 or M centrosomal staining pattern (two separate centrosomes) from panel A were counted and quantified (B). (C) U2-OS cells were first transfected with GFP-Centrin-2 and then were treated with 0.5 µM of MMC overnight. Cells were then fixed in methanol and stained with antibodies against Chibby (blue) and FancJ (red). Representative images are shown here. (**D**) Depletion of FancJ with siRNA impairs the MMC induced centrosome amplification. U2-OS cells were transfected with either Control siRNA or four different siRNAs against FancJ, treated with 0.5 µM MMC for 72 hours, fixed in methanol and then stained with antibody against γ-Tubulin. More than 300 cells were counted and the percentage of cells with more than two centrosomes was quantitated. All error bars are standard deviation obtained from three different experiments. Standard two-sided t test, *P<0.05, **P<0.01, ***P<0.001.

Because the major function of FA proteins is to repair ICL lesions and we found that FancJ has a role in regulating the normal centrosome cycle, as well as HU-induced centrosome amplification (Fig. 2C–E; supplementary material Fig. S4C), we hypothesized that FancJ connects the ICL response to centrosome amplification. To explore this idea, we first examined whether MMC could alter the centrosome staining of FancJ. As demonstrated in Fig. 1, FancJ localized to the centrosome during G1, S phase and early G2 of the cell cycle. However, during late G2 and mitosis, the centrosome staining of FancJ was dramatically reduced. Intriguingly, MMC significantly stimulated the centrosome staining of FancJ in late G2 cells (Fig. 4A). When these results were quantitated, it was found that, in non-MMC treated cells, 55% of centrosomes in late G2 cells were negative for FancJ staining and 40% had only one centrosome stain positive for FancJ (Fig. 1D, Fig. 4B). However, in MMC treated cells, 75% of the cells showed one centrosome stained positive for FancJ and 20% of both centrosomes stained positive. Furthermore, a large majority of centrosomal FancJ in MMC treated cells co-stained with the mother centrosome marker Chibby (a representative image is shown in Fig. 4C), suggesting that FancJ preferentially localized to the mother centrosome in MMC treated cells. Most importantly, depletion of FancJ with siRNA reduced the MMC-induced centrosome amplification by about 30% (Fig. 4D). This decrease was not due to cell cycle alteration in FancJ siRNA depleted cells (supplementary material Fig. S6).

Data shown in Fig. 4 strongly indicated that in addition to robustly inducing DDR, ICL inducers also enhanced the centrosome localization of FancJ during late G2, where FancJ likely promotes centrosome amplification in cells with irreparable ICL lesions.

### FancJ binds and activates PLK1 during MMC-induced centrosome amplification

**Fig. 5.**
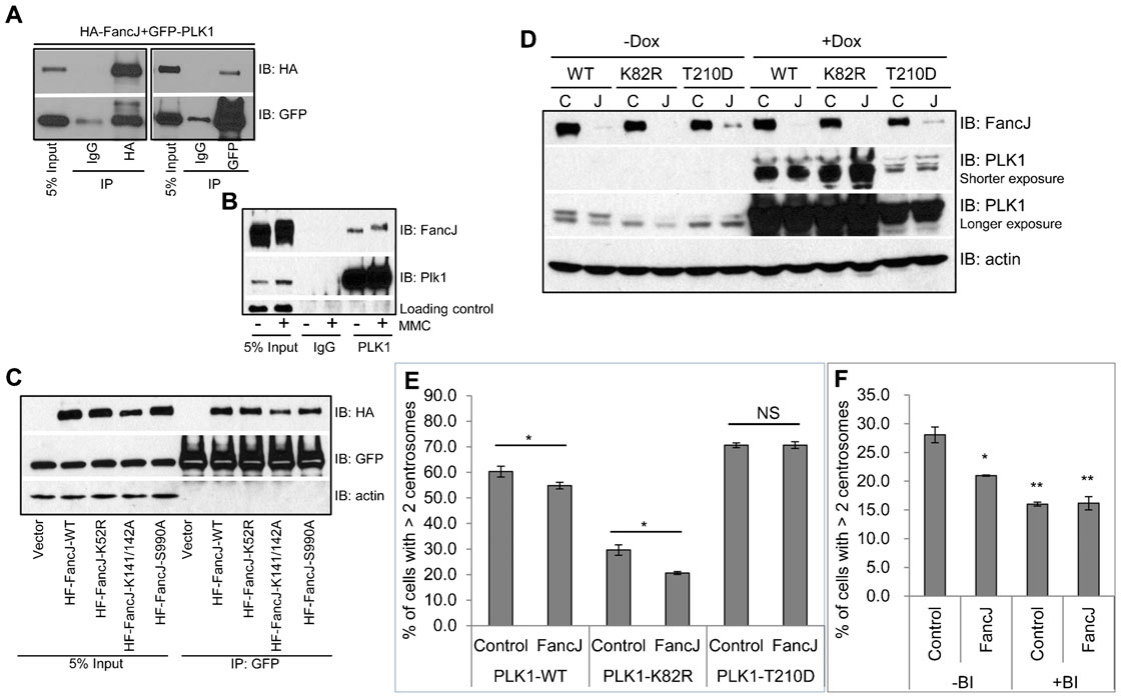
FancJ binds PLK1 and promotes the activation of PLK1 during the MMC induced centrosome amplification. (**A**) HA tagged FancJ and GFP tagged PLK1 were co-transfected into 293T cells. An equal amount of cell lysate was used for immunoprecipitation with either IgG or antibody against HA or GFP as indicated. Antibodies used for immunoblotting are indicated on the right. (**B**) 293T cells were either left untreated or treated with 0.5 µM of MMC for 24 hours. An equal amount of cell lysate was used for immunoprecipitation with either IgG or antibody against PLK1. Antibodies used for immunoblotting are indicated on the right. (**C**) Either vector or different HA tagged FancJ variants were co-transfected with GFP tagged PLK1 into 293T cells. An equal amount of cell lysate was used for immunoprecipitation with the antibody against GFP. Antibodies used for immunoblotting are indicated on the right. (**D**) Three different cell lines expressing PLK1 under control of a doxycycline (Dox)-inducible promoter were first transfected with either Control siRNA (C) or pooled siRNA against FancJ (J) and then split into two sets. Doxycycline was added to one set of the cells to induce the expression of different PLK1 variants (+Dox). 24 hours later, cells were collected and used for Western Blot analysis. Antibodies used for immunoblotting are indicated on the right. (**E**) The three Dox-regulated cell lines were first transfected with either Control siRNA (Control), or pooled siRNA against FancJ (FancJ), and then treated with doxycycline to induce the expression of different PLK1 variants. This was followed by treatment with 0.5 µM MMC for 72 hours. Cells were finally fixed in methanol and stained with antibody against γ-Tubulin. More than 300 cells were counted and the percentage of cells with more than two centrosomes was quantitated. (**F**) U2-OS cells were first transfected with either Control siRNA or siRNA against FancJ and then split into two sets. One set was treated with 0.5 µM MMC for 72 hours (−BI). In the second set, cells were first treated with 0.5 µM MMC for 12 hours and followed by the addition of 100 nM BI-2536 (+BI). Sixty hours after the addition of BI-2536, cells were finally fixed in methanol and stained with antibody against γ-Tubulin. More than 300 cells were counted and the percentage of cells with more than two centrosomes was quantitated. All error bars are standard deviation obtained from three different experiments. Standard two-sided t test, *P<0.05, **P<0.01, ***P<0.001, NS, no significance.

Because PLK1 is an important regulator of DDICA (Fig. 3D) (Inanç et al., 2010; Liu and Erikson, 2002; Lončarek et al., 2010), we wondered if FancJ regulates MMC-induced centrosome amplification through PLK1. First, we examined if FancJ interacts with PLK1. We co-transfected HA-tagged FancJ and Green Fluorescent Protein (GFP)-tagged PLK1 in 293T cells and performed co-immunoprecipitation analysis. As shown in Fig. 5A, there was a strong interaction between FancJ and PLK1. MMC treatment has no pronounced effects on the interaction of endogenous PLK1 and FancJ (Fig. 5B). FancJ was originally identified as a BRCA1 associated C-terminal Helicase; thus it is also called BACH1 or BRIP1 (Cantor et al., 2001). Lysine^52^ of FancJ is critical for its helicase activity (Cantor et al., 2001). Mutating Lysine^52^ of FancJ to alanine abolishes this activity. Phosphorylation of Serine^990^ of FancJ enhances its association with BRCA1 (Huen et al., 2010; Yu et al., 2003) while mutating Serine^990^ to alanine impairs this interaction. In addition, FancJ also binds the mismatch repair factor MutLα and mutating Lysine^141^ and Lysine^142^ to alanine abolishes this interaction (Peng et al., 2007). As shown in Fig. 5C, none of the DDR defective mutants of FancJ affected its interaction with PLK1, suggesting that the function of FancJ in regulating the centrosome cycle may be independent of its function in DDR.

Next, we tested whether FancJ regulates MMC-induced centrosome amplification through PLK1. The data in Fig. 4D indicated that depletion of FancJ decreased MMC-induced centrosome amplification. If this was due to impaired PLK1 activation, overexpressing a constitutively active PLK1 should rescue this reduction. Medema and colleagues previously generated three doxycycline (Dox)-inducible PLK1 variants in U2-OS cells: a wild-type (WT) PLK1, a kinase dead PLK1 (K82R), and a constitutively active PLK1 (T210D) (Macůrek et al., 2008). The three cell lines were first transfected with either Control siRNA or siRNA against FancJ, and subsequently the expression of PLK1 was induced with Dox. The cells were then treated with MMC for 72 hours, stained with anti-γ-Tubulin antibody to mark the centrosome and the percentage of cells with more than two centrosomes was quantified. Depletion of FancJ in all three Dox-inducible cell lines was equally efficient as in generic U2-OS cells (Fig. 5D). As shown in Fig. 5E, overexpression of either PLK1-WT or PLK1-T210D in Control siRNA transfected cells doubled the number of cells with more than two centrosomes, while overexpression of the kinase-dead PLK1 did not. This was consistent with the conclusion drawn from Fig. 3D that PLK1 promoted MMC induced centrosome amplification. Most importantly, overexpression of either PLK1-WT or PLK1-T210D, but not the kinase-dead PLK1 (PLK1-K82R), rescued the reduced centrosome amplification in FancJ siRNA transfected cells.

To further investigate the epistatic relationship between FancJ and PLK1, we examined the effects of simultaneously depleting FancJ with siRNA and inactivating PLK1 with BI-2536 on the MMC-induced centrosome amplification and compared it with that of depleting FancJ or treatment with BI-2536 alone (Fig. 5F). Addition of BI-2536 did not affect the cell cycle profile of MMC-treated cells (supplementary material Fig. S6). Consistent with data shown in Fig. 3D and Fig. 4D, BI-2536 reduced the MMC-induced centrosome amplification in Control siRNA treated cells by about 50%, while depletion of FancJ alone reduced the MMC-induced centrosome amplification by about 30%. Interestingly, simultaneous depletion of FancJ and treatment with BI-2536 reduced the MMC-induced centrosome amplification to the same extent as BI-2536 treatment alone, suggesting that FancJ is only partially responsible for the activation of PLK1 during this process. Taken together, these data strongly suggest that FancJ associates with PLK1 and promotes MMC-induced centrosome amplification through activating PLK1.

## Discussion

### Novel functions of FancJ in regulating normal centrosome cycle and ICL induced centrosome amplification

Genome stability is extremely important for animal health as its dysfunction often leads to cancer and other genetic diseases. DNA damage response (DDR) and the centrosome cycle are two of the most critical cellular processes affecting genome stability. Sporadic evidence suggests a connection between these two processes. For example, a few known key DDR factors, including the tumor suppressor BRCA1, can localize to the centrosome and play a role in regulating the centrosome cycle and in centrosome-related biology (Löffler et al., 2006; Shimada and Komatsu, 2009). It is also known that, in addition to damaging the genome, certain genotoxic stress, such as ionizing radiation (IR) and replication blockers, can induce pronounced centrosome amplification (Hut et al., 2003; Sibon et al., 2000). On the other hand, deficiency of certain centrosomal proteins, such as pericentrin (PCNT), CEP131, CEP152, and CEP164, causes DDR defects (Chaki et al., 2012; Griffith et al., 2008; Kalay et al., 2011; Staples et al., 2012).

In a recent immunofluorescence screening to identify DDR proteins that localize to the centrosome, we found that the products of six FA-related genes (out of ten tested) including FancI and FancJ localize to the centrosome. The primary functions of FA-related factors, such as FancJ, include DNA damage checkpoint and DNA repair in response to ICL lesions. Here we have uncovered novel roles of FancJ in suppressing centrosome amplification under non-stress condition and at the same time, promoting HU and MMC induced centrosome amplification (Fig. 2C–E, Fig. 4D; supplementary material Fig. S4). Though we did not detect pronounced cell cycle changes when FancJ is depleted with siRNA by flow cytometry analysis (supplementary material Fig. S6), we can not completely rule out the possibility that some of our observations could be due to subtle cell cycle alterations that failed to be detected by flow cytometry. On the other hand, these two seemingly paradoxical functions of FancJ in the centrosome biogenesis may be consistent with its function as a tumor suppressor. Under non-stress condition, FancJ suppresses the centrosome amplification which could promote tumorigenesis (Basto et al., 2006). However when a cell experiences extensive DNA damage, it needs to be eliminated from the organism otherwise it could potentially initiate tumor development (Nigg and Stearns, 2011; Sibon et al., 2000).

Mechanistically, we still do not know how FancJ regulates the normal centrosome cycle. FancJ, also called BACH1/BRIP1, is part of the BRCA1 B-complex (Cantor et al., 2001; Wang et al., 2007). BRCA1 has well-established functions in regulating centrosome biogenesis (Kais and Parvin, 2008). Whether under non-stress conditions FancJ regulates centrosome biogenesis through BRCA1 certainly warrants further investigation. Since we found that FancJ can bind PLK1 and overexpression of either wild-type or the constitutively active, but not the kinase-dead, PLK1 rescues the reduced centrosome amplification in FancJ deficient cells (Fig. 5), we propose that FancJ acts upstream of PLK1 and stimulates its activation during DDICA. Whether FancJ activates PLK1 in the cytoplasm or at the centrosome needs further investigation. Alternatively, FancJ may help to recruit or retain PLK1 at the centrosome. DNA damage checkpoint and DNA repair often take place within minutes or hours after sensing genotoxic stress. DDICA, on the other hand, is a delayed response. As shown in Fig. 3B, even after 48 hours of MMC treatment, there is only a very moderate increase of cells with amplified centrosomes. Only around 60 hours after MMC treatment, do a significant fraction of cells show amplified centrosomes. FancJ has a well-established function in ICL-induced checkpoint and repair functions (Cantor and Guillemette, 2011; Hiom, 2010). Here we showed that FancJ also plays a role in regulating the DDICA. Therefore, FancJ is implicated in both the early as well as the late event of ICL response, suggesting that it is the potential liaison between them.

We have examined the role of two FA-related factors that localize to the centrosome in the regulation of the centrosome biogenesis and found that only the deficiency of FancJ affects the centrosome cycle. The interesting question becomes why FancI localizes to the centrosome. We detected centrosome localization of FancI with three different antibodies against endogenous FancI, as well as with anti-FLAG antibody in cells expressing FLAG-FancI (Fig. 1; supplementary material Fig. S2). Conversely, siRNA depletion of FancI reduces its centrosome staining (supplementary material Fig. S3A,B). Therefore we think that the centrosome localization of FancI is less likely an artifact caused by the antibodies. FancI can be phosphorylated (Ishiai et al., 2008; Matsuoka et al., 2007; Sareen et al., 2012), and the candidate kinase such as CDKs and ATM/ATR are also found at the centrosome (supplementary material Table S1) (Doxsey et al., 2005; Shimada and Komatsu, 2009). As discussed above, we speculate that FancI may be phosphorylated at the centrosome by CDKs and/or ATM/ATR. Though its role in centrosome biogenesis is still unknown, depletion of FancM with siRNA also induces centrosome amplification (Collis et al., 2008). Together, these evidences suggest that there may be a two-way regulation between FA-related factors and the centrosome. On the one hand, some of the FA proteins regulate centrosome biogenesis, especially DDICA, in addition to their roles in repairing the ICL lesions. On the other hand, FA proteins may be phosphorylated at the centrosome and/or FA sub-complexes are assembled at the centrosome (Medhurst et al., 2006).

### The centrosome biogenesis function of FancJ likely contributes to its physiological and pathophysiological functions

Fanconi Anemia (FA) is a hereditary disease characterized by clinical features such as bone marrow failure, congenital developmental defects, and predisposition to and early onset of a variety of cancers, including acute myeloid leukemia (AML), squamous cell carcinoma of the head and neck, and other blood and solid tumors (Alter, 1996; Alter, 2003). Loss-of-function mutations in *FancJ* genes are found in FA patients as well as in breast cancer patients suggesting that FancJ is a *bona fide* tumor suppressor (Cantor and Guillemette, 2011; Suhasini and Brosh, 2013). The pathological phenotypes seen in FancJ mutation carriers are likely due to their critical functions in a variety of cell types including different adult stem cells (Kottemann and Smogorzewska, 2013). Centrosome amplification is commonly found in all these cancers (Nigg, 2006). Centrosome has also recently been linked to stem cell biology (Nigg and Raff, 2009). Our data demonstrate that FancJ suppresses the centrosome amplification under a non-stress condition and promotes centrosome amplification when cells encounter genotoxic stress. Both processes might contribute to the tumor suppressing function of FancJ. Therefore, the role of FancJ in both the normal centrosome cycle as well as DDICA could contribute to its physiological and pathophysiological function in both FA patients and breast cancer patients.

## Materials and Methods

### Plasmids

Plasmids expressing C-terminal HA and FLAG-tagged FancJ-WT, FancJ-K52R, FancJ-K141/142A, and FancJ-S990A are generously provided by Dr Sharon B. Cantor. FLAG-tagged FancI and GFP-tagged Centrin-2 are generously provided by Drs Stephen Elledge and Alexey Khodjakov, respectively. For GFP-PLK: PLK1 cDNA was first cloned into pENTR/D-TOPO (purchased from Invitrogen) and then fused with a Gateway destination vector with N-terminal GFP tag generously provided by Dr Jianping Jin (University of Texas – Houston Medical School).

### siRNAs

Fanc-I2 (Invitrogen, TTAACAAGGTGTCCACACAGCTGCC) and Fanc-I3 (Invitrogen, GCTGGTGAAGCTGTCTGGTTCTCA) (Smogorzewska et al., 2007), FancJ-A (Dharmacon, AGTCAAGAGTCATCGAATA), FancJ-B (Dharmacon, GATAGTATGGTCAACAATA), FancJ-C (Dharmacon,TAACCCAAGTCGCTATATA), FancJ-A (Dharmacon, GTGCAAAGCCTGGGATATA), pooled FancJ siRNA (Dharmacon, mixture of equal amount of FancJ-A to -D). All FancJ siRNA are ON-TARGETplus. ON-TARGETplus siCONTROL Non-targeting pool (Dharmacon, D-001810-10-20) was used as a negative control for all siRNA transfections. Human cells were transfected with 50 nM siRNA twice using RNAiMAX (Invitrogen).

### Chemicals

Hydroxyurea (Sigma, H8627), BI2536 (Selleck, S1109), mitomycin C (Research Products International, M92010-0.01), doxycycline (Research Products International, D43020).

### Antibodies

Mouse GFP (Clontech, clone JL-8). Rabbit GFP (Invitrogen, A-11122). HA (Convance, MMS 101P). FLAG (Sigma, clone M2). Actin (Santa Cruz, sc-1616). PLK1 (Millipore, 05-844) and PLK1 (Bethyl, A300-251A). Centrin 2 (Santa Cruz, sc-27793-R). γ-Tubulin (Sigma, T5326 and T3195). GAPDH (Bethyl, A300-641A). Chibby (Santa Cruz, sc-101551). FancJ (Bethyl, A300-561A). Following antibodies are generously provided by the Fanconi Anemia Research Fund: FancA, FancB, FancG, FancI1, FancI2, FancM1, and FancM2. Drs Stephen Elledge and Lei Li generously provided FancI3 and FancM3, respectively.

### Cell lines and cell culture

293T, Hs587T, HeLa, and U2-OS cells were purchased from ATCC. All cells were grown in D-MEM supplemented with 10% fetal bovine serum (FBS) and Penicillin and Streptomycin. All cells were cultivated at 37°C in a humidified incubator with 5% CO_2_.

### Cell lysis and immunoprecipitation

For whole cell lysates (WCL), cells were lysed in NETN-150 buffer (20 mM Tris-HCl, pH 8.0; 150 mM NaCl; 1 mM EDTA; 0.5% NP-40) containing a cocktail of phosphatase and protease inhibitors (Sigma). For immunoprecipitation: equal amount of cell lysate were incubated with primary antibody and protein A Sepharose CL-4B beads (GE Healthcare, 17-078-01) with rotation at 4°C overnight.

### Centrosome staining

Cells grown on a glass cover-slip (Fisher, 12-544-10) were washed twice with 1× PBS and then permeabilized in ice cold 0.5% Triton X-100 in 1× PBS for 2 min. Cells were then washed twice with 1× PBS and fixed with 100% methanol at −20°C for 5 min. Cells were washed twice with 1× PBS. Cells were incubated in 1% gelatin at room temperature for 10 min. Cells were washed twice with 1× PBS. Cells were incubated in 0.02 M glycine at room temperature for 3 min. Cells were washed twice with 1× PBS. Cells were incubated in primary antibody at room temperature for one hour. Primary antibody is prepared in 1× PBS with 1% BSA. Cells were washed twice with 1× PBS. Cells were incubated in secondary antibody in the dark at room temperature for one hour. Secondary antibody is prepared in 1× PBS with 1% BSA. Cells were washed twice with 1× PBS. Cells finally were mounted using Prolong Gold antifade reagent with DAPI (Invitrogen, P36931). All the images except the triple stainings were acquired using an Olympus Fluorescent Microscope, BX60, equipped with a Nikon DS-Qi1camcera and analyzed with Nikon NIS-Element software. All the images taken with BX60 are from a single focal plane. For the triple staining, GFP-Centrin-2 transfected cells were treated with a mixture of Cy3- and Cy5-conjugated mouse and rabbit secondary antibodies (Jackson ImmunoResearch Laboratories). The triple staining images were acquired with an Olympus FluoView 500 Laser Scanning Confocal Microscope with six laser lines and they are the projections of z stacks.

For the quantification of data shown in supplementary material Fig. S3B,D, the staining intensities of both γ-Tubulin and FancI (supplementary material Fig. S3B) or FancJ (supplementary material Fig. S3D) at the centrosomes were measured in thirty seven to forty cells of both Control siRNA transfected as well as FancI or FancJ siRNA transfected cells. The intensity of each centrosome staining of FancI and FancJ was normalized against the intensity of the corresponding γ-Tubulin. The data was processed and plotted using Prism GraphPad 6.02. The error bars are the Tukey's confidence limits.

## Supplementary Material

Supplementary Material
