# Supplementary material for: FancJ regulates interstrand crosslinker induced centrosome amplification through the activation of polo-like kinase 1
Source: Biol Open. 2013 Aug 6;2(10):1022–31. doi: 10.1242/bio.20135801 (PMC3798185; doi:10.1242/bio.20135801)
Supplement: Supplementary Material [file supp_2_10_1022__index.html]

FancJ regulates interstrand crosslinker induced centrosome amplification through the activation of polo-like kinase 1 — FancJ regulates interstrand crosslinker induced centrosome amplification through the activation of polo-like kinase 1 — Supplementary Material 

# FancJ regulates interstrand crosslinker induced centrosome amplification through the activation of polo-like kinase 1

## bio.20135801 Supplementary Material

**Files in this Data Supplement:**

- Supplementary Material - Jianqiu Zou et al. doi: 10.1242/bio.20135801
