# Supplementary material for: FancJ regulates interstrand crosslinker induced centrosome amplification through the activation of polo-like kinase 1
Source: Biol Open. 2013 Aug 6;2(10):1022–31. doi: 10.1242/bio.20135801 (PMC3798185; doi:10.1242/bio.20135801)
Supplement: Supplementary Material [file supp_bio.20135801_bio.20135801-s1.pdf]

Supplementary Material

Jianqiu Zou et al. doi: 10.1242/bio.20135801

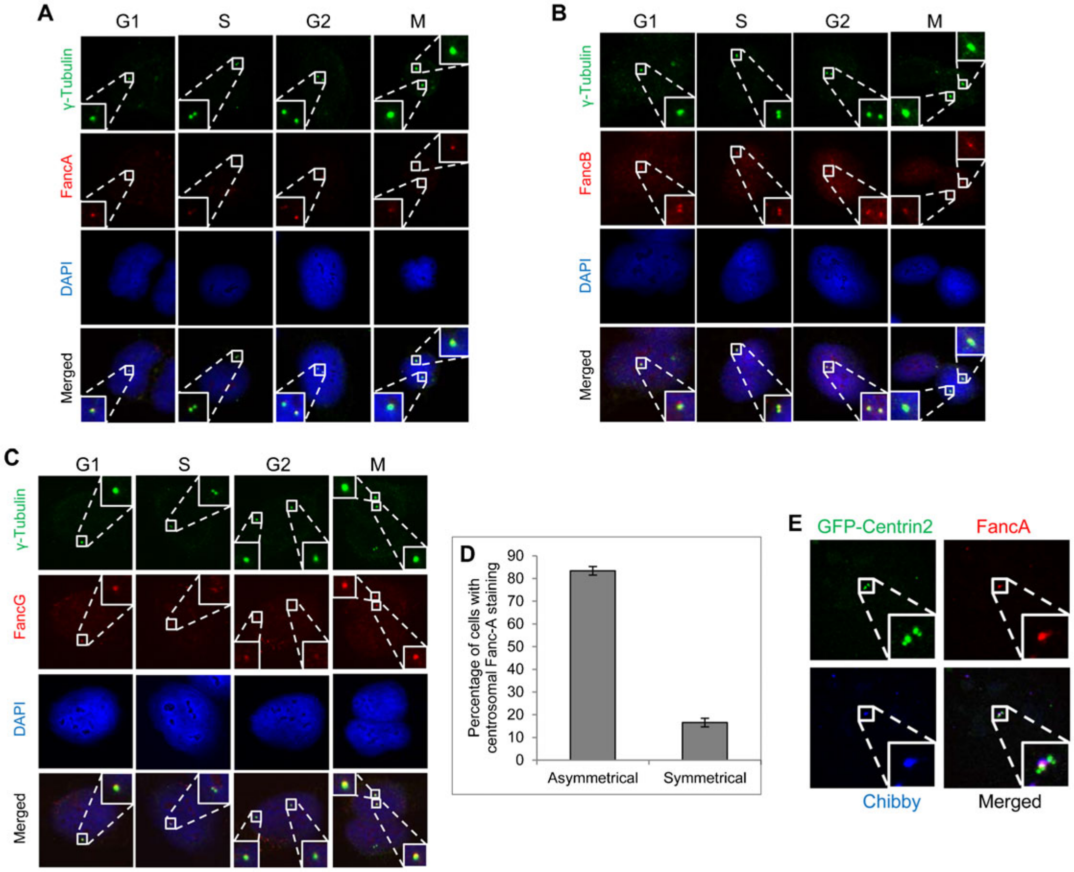

Fig. S1. Three other Fanconi Anemia (FA) related proteins, FancA, FancB, and FancG, localize to the centrosome. U2-OS cells were fixed in methanol and co-stained with antibodies against γ-Tubulin (green) and the corresponding FA-related protein (red) as indicated, FancA (**A**), FancB (**B**), and FancG (**C**). Nuclei were stained with DAPI (blue). (**D**) U2-OS cells were stained with antibodies against γ-Tubulin (green) and FancA (red). More than 100 cells with a G2 or M centrosomal staining pattern were analyzed. A cell was counted as having symmetrical centrosome staining when both of the separated centrosomes stained positive for FancA. A cell with asymmetrical centrosome staining had only one of the two separated centrosomes stained positive for FancA. All error bars are the standard deviations obtained from three different experiments. (**E**) U2-OS cells were first transfected with GFP-Centrin-2 then fixed in methanol and stained with antibodies against Chibby (blue) and FancA (red). Representative images are shown.

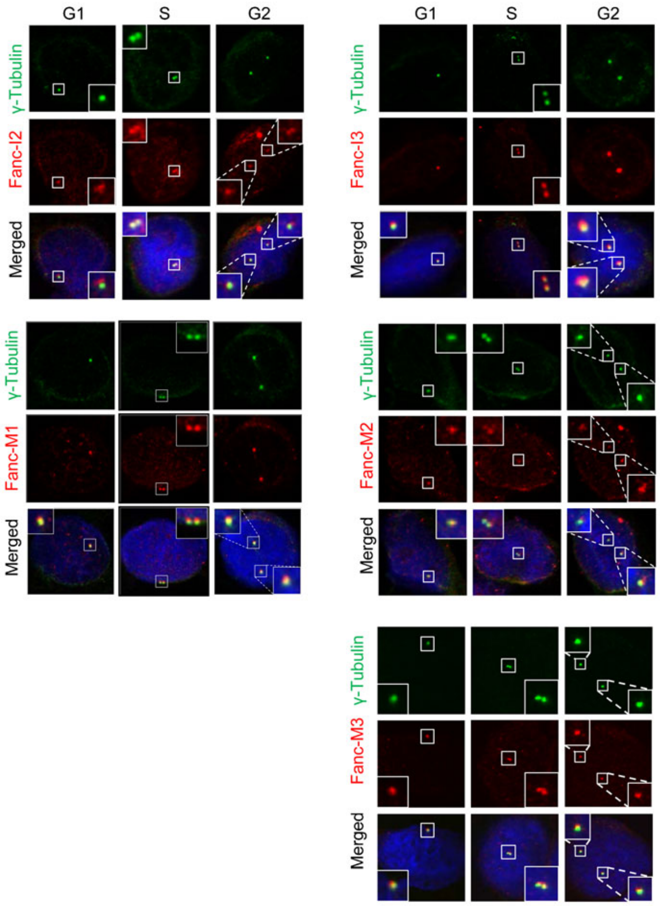

Fig. S2. Multiple antibodies against FancI and FancM stain positive of centrosome. U2-OS cells were fixed in methanol and stained with antibodies against γ-Tubulin (green) and either FancI (red, top panels) or FancM (red, middle and bottom panels). Nuclei were stained with DAPI (blue). Fanc-I2 and Fanc-I3 are the second and the third antibodies against FancI. Fanc-M1, Fanc-M2 and Fanc-M3 are three different antibodies against FancM.

Biology Open

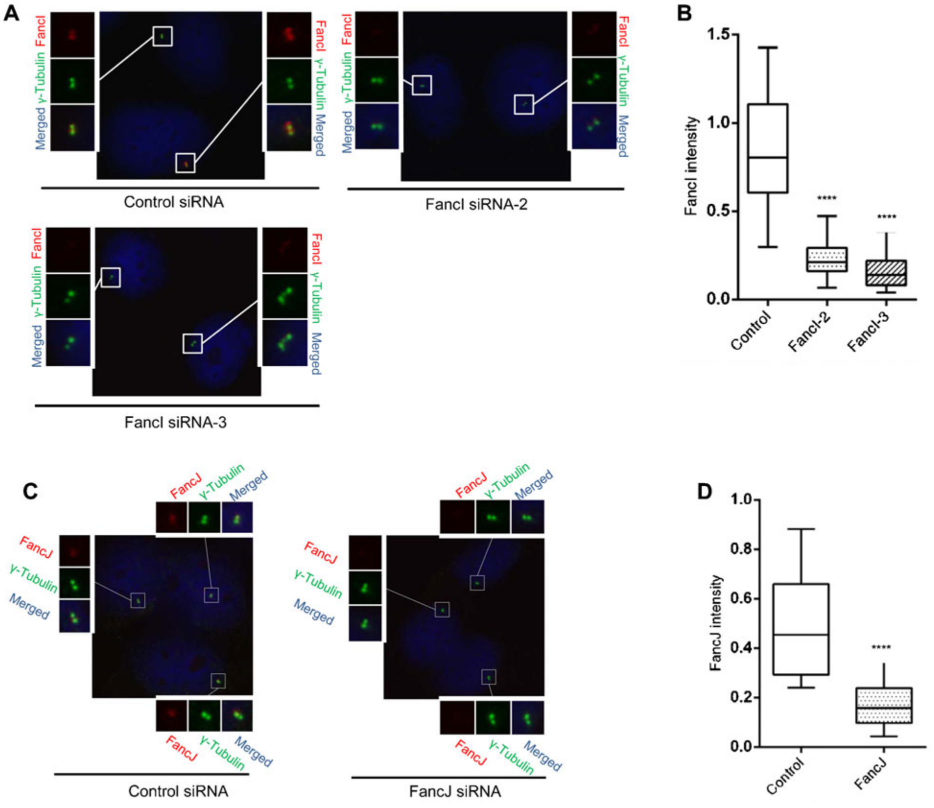

Fig. S3. Depletion of FancI or FancJ with siRNA reduces their centrosome staining. U2-OS cells were first transfected with either Control siRNA or siRNA against FancI (**A**,**B**) or FancJ (**C**,**D**). Cells were then fixed in methanol and stained with antibody against γ-Tubulin (green) and FancI (red) (A) or FancJ (red) (C). The intensity of FancI (B) or FancJ (D) centrosome staining in around forty cells was quantified and normalized against the intensity of γ-Tubulin. Error bars are the Tukey's confidence limits. ****$P \ll 0.001$.

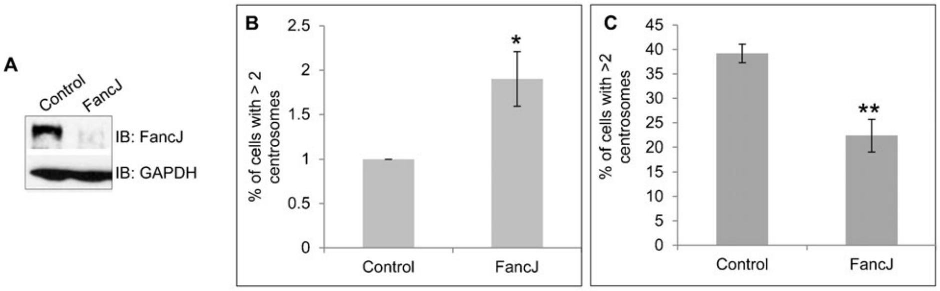

Fig. S4. Depletion of FancJ with siRNA in Hs587T cells impairs centrosome biogenesis. Hs587T cells were transfected with either Control siRNA or pooled siRNA against FancJ. Cells were then split into three sets. One set was used for Western Blot analysis to monitor the siRNA knockdown efficiency (**A**). Antibodies used for immunoblotting are indicated on the right. The second set was fixed in methanol and stained with antibody against γ-Tubulin (**B**). The third set was first treated with 16 mM HU for 68 hours and then fixed in methanol and stained with antibody against γ-Tubulin (**C**). More than 300 cells were counted and the percentage of cells with more than two centrosomes was quantitated. All error bars are standard deviations obtained from three different experiments. Standard two-sided t test, *$P<0.05$, **$P<0.01$, ***$P<0.001$.

Biology Open

Biology Open

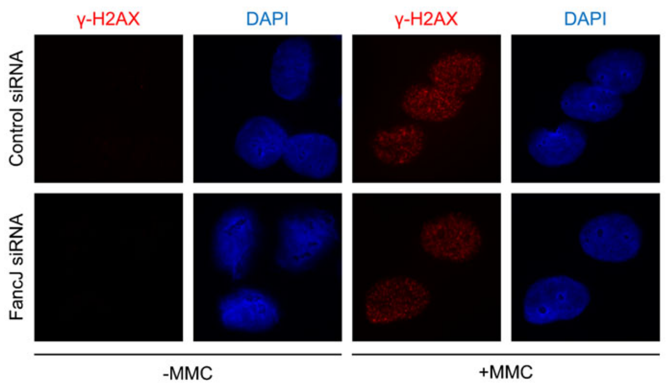

Fig. S5. Depletion of FancJ does not induce pronounced DNA damage. U2-OS cells were transfected with either Control siRNA or siRNA against FancJ. Cells were either left untreated or treated with 0.5 µM MMC and then fixed in methanol and stained with antibody against γ-H2AX (red). Nuclei were stained with DAPI (blue).

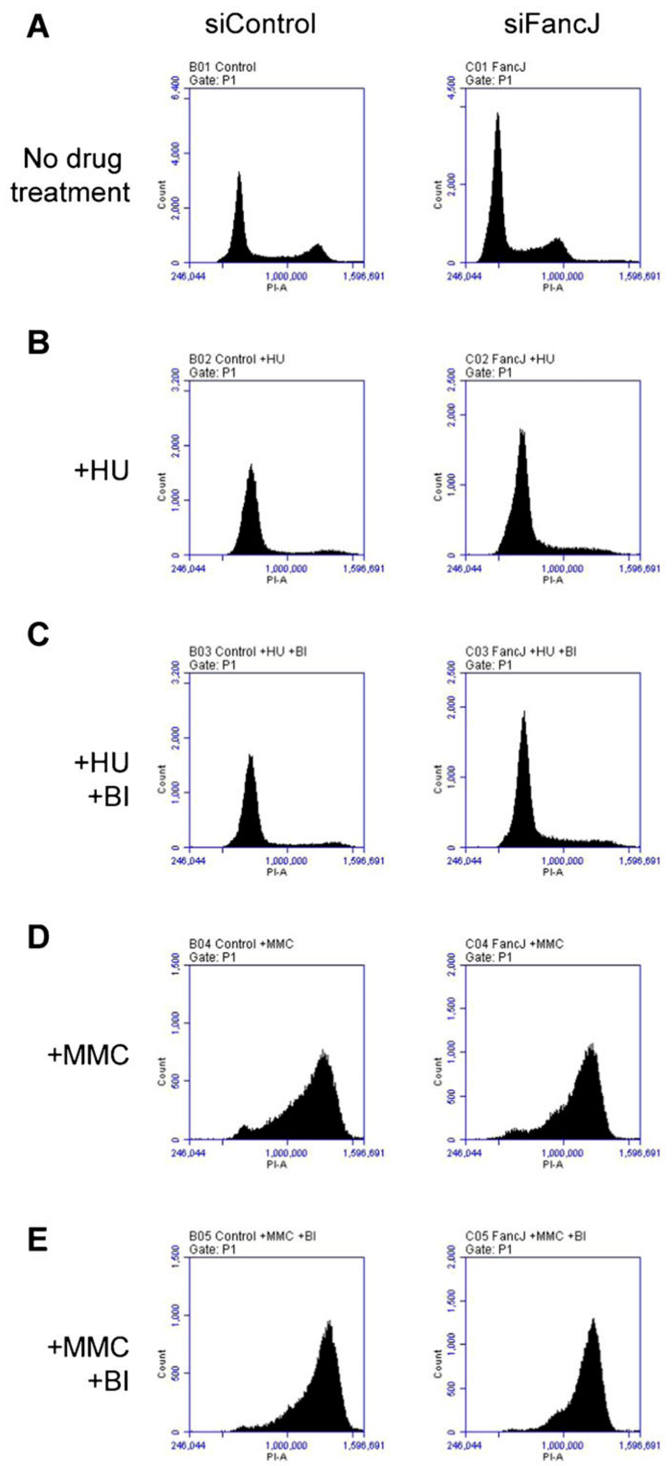

Fig. S6. Cell cycle analysis. U2-OS cells were first transfected with either Control siRNA or siRNA against FancJ and then split into five sets. The first set of cells was stained with propidium iodide (PI) directly (**A**). The second set of cells was treated with 16 mM HU for 72 hours and stained with PI (**B**). For the third set of cells, 12 hours after the addition of 16 mM HU, 100 nM of BI-2536 was added. Sixty hours later, cells were stained with PI (**C**). The fourth set of cells was treated with 0.5 µM MMC for 72 hours and stained with PI (**D**). For the fifth set of cells, 12 hours after the addition of 0.5 µM MMC, 100 nM of BI-2536 was added. Sixty hours later, cells were stained with PI (**E**). All PI stained cells were analyzed by a flow cytometer.

Biology Open

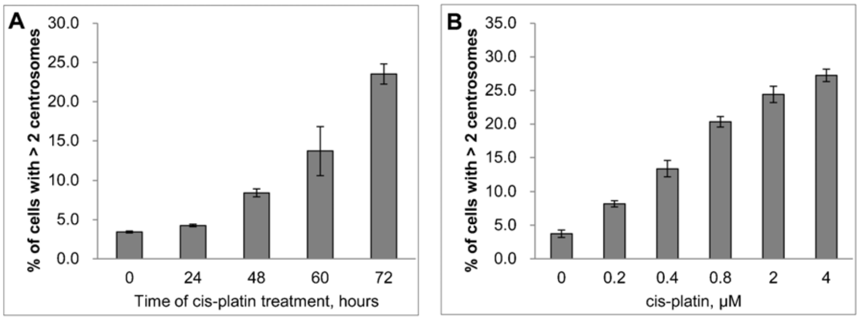

Fig. S7. Cis-platin induces pronounced centrosome amplification. (**A**) Time-course experiment. U2-OS cells were first treated with 2 µM cis-platin. At the indicated time, cells were fixed in methanol and stained with antibody against γ-Tubulin. (**B**) Dose response experiment. 72 hours after treatment with different concentrations of cis-platin, U2-OS cells were fixed in methanol and stained with antibody against γ-Tubulin. More than 300 cells were counted and the percentage of cells with more than two centrosomes was quantitated.

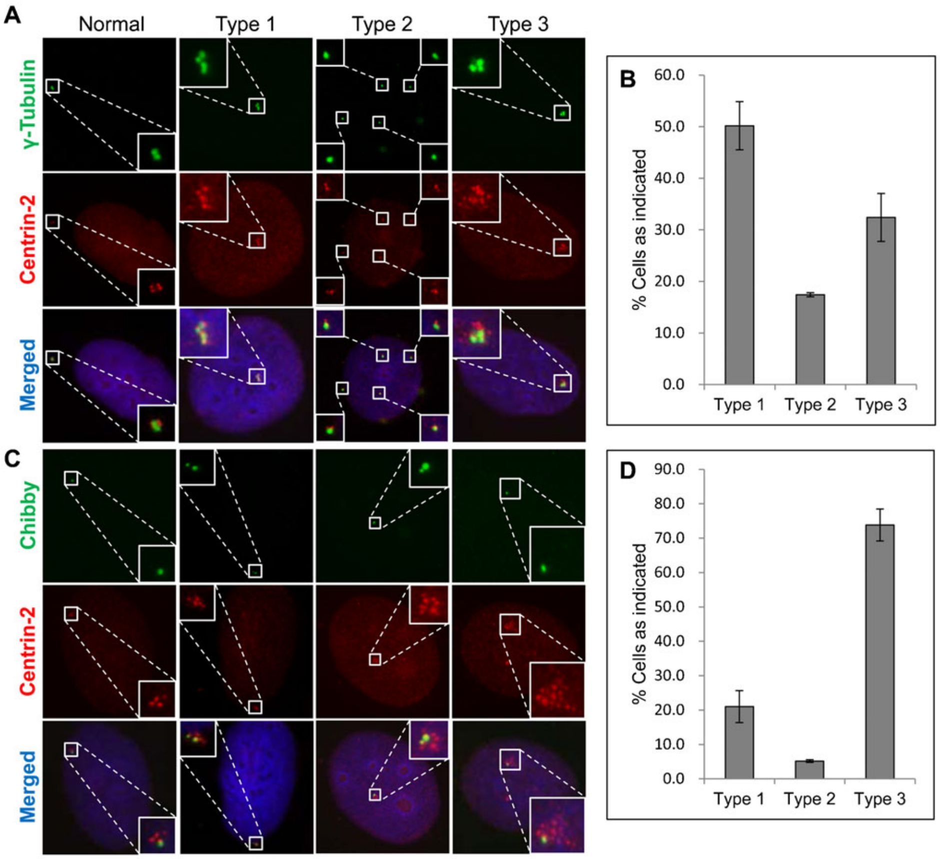

Fig. S8. MMC induced centrosome amplification. U2-OS cells were first treated with 0.5 µM MMC for 72 hours and then fixed in methanol and stained with antibodies against γ-Tubulin (green) and Centrin-2 (Red) (**A**,**B**), or antibodies against Chibby (green) and Centrin-2 (Red) (**C**,**D**). Nuclei were stained with DAPI (blue). The detail of the classification can be found in the text. More than fifty cells with amplified centrosomes were counted. All error bars are standard deviations obtained from three different experiments.

Table S1. Known DNA damage and DNA replication related proteins that localize to centrosome.

	Reference
ATM	(Brown and Costanzo, 2009; Smith et al., 2009; Zhang et al., 2007)
ATR	(Zhang et al., 2007)
ATRIP	(Zhang et al., 2007)
CHK1	(Zhang et al., 2007)
CHK2	(Zhang et al., 2007)
DNA-PK	(Yang et al., 2003; Zhang et al., 2007)
BRCA1	(Sankaran et al., 2006; Shimada et al., 2009; Starita et al., 2004)
BRCA2	(Niwa et al., 2009)
NBS1	(Shimada et al., 2009)
ORC1	(Ferguson et al., 2010; Hemerly et al., 2009)
ORC2	(Prasanth et al., 2004)
ORC subunit 1 to 5	(Stuermer et al., 2007)
MCM5	(Ferguson et al., 2010)
MCM subunit 2 to 7	(Stuermer et al., 2007)
p53	(Tritarelli et al., 2004)
PARP1	(Augustin et al., 2003)
PARP3	(Augustin et al., 2003)
RAD51	(Lesca et al., 2005)
Tankyrase (telomeric PARP)	(Smith and de Lange, 1999)
TopBP1	(Bang et al., 2011)
